# Eyesight and School Life

**Published:** 1895-12

**Authors:** 


Eyesight and School Life. By Simeon Snell, F. R. C. S., Ed., Ophthalmic Surgeon to the Sheffield General Infirmary and to the School for the Blind; Lecturer on Diseases of the Eye at the Sheffield Medical School; Consulting Ophthalmic Surgeon to the Rotherham Hospital; Author of The Electro-magnet in Ophthalmic Surgery, Miner's Nystagmus, etc. With illustrations. Duodecimo, pp. xii—70. Price, 63 cents. Bristol: John Wright & Co. 1895.

Mr. Snell has somewhat extended a popular lecture which he originally delivered before an association of teachers, and has placed it before a larger audience in the form of this little book. The subject matter embraces the consideration, in nontechnical language, of the accommodation and refraction of the eye; refractive errors, their causes and effects; and the hygiene of the school room, so far as it appertains to lighting, desks and seating of pupils and the proper positions for reading and writing. The author then adds some useful suggestions regarding hours of study, physical exercise, duties of parents and duties of teachers, and concludes with remarks on the results of proper correction of errors of refraction.

This little work is very practical and is to be commended to teachers, parents and others who are concerned with school life.

